# *IFITM5* pathogenic variant causes osteogenesis imperfecta V with various phenotype severity in Ukrainian and Vietnamese patients

**DOI:** 10.1186/s40246-019-0209-3

**Published:** 2019-06-03

**Authors:** Lidiia Zhytnik, Katre Maasalu, Binh Ho Duy, Andrey Pashenko, Sergey Khmyzov, Ene Reimann, Ele Prans, Sulev Kõks, Aare Märtson

**Affiliations:** 1 Department of Traumatology and Orthopeadics, University of Tartu, Puusepa 8, 51014 Tartu, Estonia; 2 Clinic of Traumatology and Orthopeadics, Tartu University Hospital, Puusepa 8, 51014 Tartu, Estonia; 3 Hue University of Medicine and Pharmacy, Hue University, Hue, Vietnam; 4 Department of Pediatric Orthopedics, Sytenko Institute of Spine and Joint Pathology, AMS Ukraine, Pushkinska 80, Kharkiv, 61024 Ukraine; 5 Centre of Translational Medicine, University of Tartu, Ravila 14a, 50411 Tartu, Estonia; 6 Department of Pathophysiology, University of Tartu, Ravila 19, 50411 Tartu, Estonia; 7 Perron Institute for Neurological and Translational Science, QEII Medical Centre, Nedlands, WA Australia

**Keywords:** Osteogenesis imperfecta, *IFITM5*, Type V, Hyperplastic callus, Bone fragility

## Abstract

**Background:**

Osteogenesis imperfecta (OI) covers a spectrum of bone fragility disorders. OI is classified into five types; however, the genetic causes of OI might hide in pathogenic variants of 20 different genes. Often clinical OI types mimic each other. This sometimes makes it impossible to identify the OI type clinically, which can be a risk for patients. Up to 90% of OI types I–IV are caused by pathogenic variants in the *COL1A1/2* genes. OI type V is caused by the c.-14C > T pathogenic variant in the 5′UTR of the *IFITM5* gene and is characterized by hyperplastic callus formation and the ossification of interosseous membranes.

**Results:**

In the current study, we performed *IFITM5* 5′UTR region mutational analysis using Sanger sequencing on 90 patients who were negative for *COL1A1*/*2* pathogenic variants. We also investigated the phenotypes of five patients with genetically confirmed OI type V. The proportion of OI type V patients in our cohort of all OI patients was 1.48%. In one family, there was a history of OI in at least three generations. Phenotype severity differed from mild to extremely severe among patients, but all patients harbored the same typical pathogenic variant. One patient had no visible symptoms of OI type V and was suspected to have had OI type IV previously. We also identified a case of extremely severe hyperplastic callus in a 15-year-old male, who has hearing loss and brittleness of teeth.

**Conclusions:**

OI type V is underlined with some unique clinical features; however, not all patients develop them. The phenotype spectrum might be even broader than previously suspected, including typical OI features: teeth brittleness, bluish sclera, hearing loss, long bones deformities, and joint laxity. We suggest that all patients negative for *COL1A1*/*2* pathogenic variants be tested for the presence of an *IFITM5* pathogenic variant, even if they are not expressing typical OI type V symptoms. Further studies on the pathological nature and hyperplastic callus formation mechanisms of OI type V are necessary.

## Background

Osteogenesis imperfecta (OI) covers a spectrum of rare, genetic connective tissue disorders, outlined by bone fragility, skeletal deformations, and recurrent fractures [1]. Typical symptoms of OI include blue sclera, dentinogenesis imperfecta (DI), hearing loss, and joint laxity [2–4].

The genetic spectrum of OI is composed of 20 different OI genes [5–7]. Up to 90% of OI cases arise from the heterozygous pathogenic variants of the *COL1A1* (OMIM 120150) and *COL1A2* (OMIM 120160) genes, which code for collagen type I α1, 2 chains [8]. One more gene representing autosomal dominant OI is the *IFITM5* (OMIM 610967) gene, which codes for a bone-restricted ifitm-like protein (BRIL) [9]. Up to 5% of individuals from OI population harbor pathogenic variants in the *IFITM5* gene [10]. The remaining OI cases are caused by pathogenic variants in the recessive OI genes [7, 11–23].

Genetic heterogeneity is reflected in the broad variability of OI phenotypes. In 1979, David Sillence classified OI based on the clinical severity as follows: type I—non-deforming OI with blue sclera; type II—lethal OI; type III—severe deforming OI; and type IV—variable OI without blue sclera [4]. In 2000, based on histological and radiological evidences, Glorieux et al. proposed the existence of an additional variant, OI type V [24]. This particular OI type differed due to its mesh-like pattern of lamellation, hyperplastic callus (HPC) formation, and calcification of the interosseous membrane in the forearm [24–26]. Patients with OI type V are described to mimic symptoms of OI type IV with moderate severity of long-bone deformities, vertebral compression fractures, scoliosis, and the same number of fractures. However, unlike type IV, patients with OI type V were claimed not to develop DI or blue sclera [25].

Recent studies have introduced variability in the OI type V phenotypes [25, 27–30]. The spectrum of OI type V phenotypes has been expanded with phenotypes mimicking OI type I and III and [31]. Despite phenotype variations, all patients with OI type V harbor the same genetic change—a pathogenic variant in the 5′ untranslated (UTR) region of the *IFITM5* gene, c.-14C > T [32, 33].

Neither the physiological function of the *IFITM5* gene product, BRIL protein, nor the effect of the mutated protein is fully understood. As it is known, c.-14C > T substitution in the 5′UTR region of the *IFITM5* gene creates a new upstream start codon and causes the inclusion of five additional amino acids to the N-termini of the BRIL protein (MALEP) [34]. However, the exact pathological mechanism of OI type V remains unclear; it was hypothesized that the MALEP-IFITM5 acts in a neomorphic manner, gaining a new molecular function [35].

Despite the genetic cause of OI type V first being published in 2012, the discovery of patients harboring the c.-14C > T *IFITM5* variant is still rare, and many of them do not develop traits characteristic of OI type V [31–33]. Thus, the description of phenotypes in patients of OI type V is of particular interest, as it enriches our understanding of phenotypical variability and approaches to the diagnosis of OI type V.

In this study, we have screened for the presence of a c.-14C > T *IFITM5* pathogenic variant in a cohort of 90 unrelated Estonian, Ukrainian, and Vietnamese OI patients negative for collagen type I pathogenic variants with diverse OI phenotypes. Herein, we report the phenotypical characteristics captured during the screening of five OI type V patients with identical c.-14C > T pathogenic variants in the *IFITM5* gene and phenotypes of different severity. Also, for the first time, we report a case of an OI type V patient with extremely severe callus and hearing loss.

The management and diagnosis of OI is performed by clinical geneticists. OI type V is one of the rarest OI types, as such, is often misdiagnosed. We believe that sharing the data from patients with rare OI types is an important contribution to the field. In many countries, not all patients undergo genetic testing and OI is often diagnosed based on phenotypical features. This article illustrates the high degree of variation in the clinical symptoms of OI type V. Additionally, it highlights the critical importance of identifying the specific variant at hand to develop an appropriate treatment strategy and counseling for family planning.

## Materials and methods

The study was performed using patients from the OI biobank of the Clinic of Traumatology and Orthopedics from the University of Tartu, Estonia (UT OI biobank). The UT OI biobank consists of 237 OI families from Estonia, Ukraine, and Vietnam, including 337 samples from OI affected individuals and their healthy relatives. Informed written consent was collected from all subjects and controls, or their legal representatives, prior to their participation in the study. The study was conducted in accordance with the Helsinki Declaration and authorized by the Ethical Review Committee on Human Research of the University of Tartu (Permit no. 221/M-34), the Ethical Review Board of Hue University Hospital (approval No. 75/CN-BVYD), and the Sytenko Institute of Spine and Joint Pathology of the Ukrainian Academy of Medical Sciences. Details of the patient recruitment process are described elsewhere. [36–38]

For the current study, we used a cohort of 90 unrelated OI patients negative for *COL1A1*/*2* pathogenic variants. Patients were previously diagnosed with OI of different severity (Sillence classification, types I–IV) based on clinical features. Only one patient was suspected to have OI type V due to significant, visible HPC.

To capture a typical pathogenic variant causing OI type V, a cohort of selected patients were screened for the presence of a c.-14C > T pathogenic variant in the *IFITM5* gene. Focusing on the mutational analysis of the specific locus, we have selected Sanger sequencing of the 5′UTR region of the *IFITM5* gene to be the most accurate approach to meet the needs of this study. Genomic DNA extraction was performed from the ethylenediaminetetraacetic acid (EDTA)-preserved blood samples using the Gentra Puregene Blood Kit (QIAGEN, Germany) according to the manufacturer’s protocol and stored at − 80 °C.

DNA samples were amplified using a PCR with a specially designed primer pair covering the 5′UTR region and exon 1 of the *IFITM5* gene.

The PCR reaction was performed with 5x HOT FIREPol® Blend Master Mix Ready to Load (Solis BioDyne, Estonia). PCR reaction was performed with a Thermal Cycler (Applied Biosystems, USA) PCR machine. Additional details on the PCR procedures are available from the authors upon reasonable request.

Amplified PCR products were electrophoresed through a 1.5% agarose gel. PCR products were purified with Exonuclease I and Shrimp Alkaline Phosphatase (Thermo Fisher Scientific, USA). Sanger sequencing reactions were performed from purified PCR fragments using BigDye® Terminator v3.1 Cycle Sequencing Kit (Applied Biosystems, USA). Reactions were processed on the ABI3730xl instrument.

Sequence reads were analyzed with Sequence Scanner v1.0 of Applied Biosystems and aligned to the RefSeq sequence NM_001025295.1. Sequence analysis and pathogenic variant identification was performed with Mutation Surveyor DNA variant analysis software (Softgenetics, USA). Presence of a c.-14C > T variant was controlled in a screened cohort. Predictions for the pathogenicity and functional impact of the 5′UTR variants were provided with Mutation Taster. All analyses were conducted at the University of Tartu (Estonia). Sequencing data is available from the authors upon reasonable request.

## Results

### Results of mutational analysis

According to clinical diagnosis, out of the 90 unrelated OI patients screened, OI was suspected only in one case (1.11%). However, after screening of the 90 unrelated patients, we have identified four patients (4.44%) harboring a heterozygous c.-14C > T variant in the 5′UTR region of the *IFITM5* gene. Three patients came from the Ukrainian OI cohort. One patient came from Vietnamese cohort. There were no patients with OI type V among the Estonian OI population. Parents of three affected patients did not harbor pathogenic variants, which confirmed the de novo nature of the pathogenic variant. Only one patient had a family history of OI, whose mother was a carrier of a c.-14C > T heterozygous variant in the *IFITM5* gene also. The overall prevalence of OI type V in the UT OI biobank was 5/337 (1.48%) (Table 1).

**Table 1:** Phenotype characteristics of OI type V patients

	Patient 1	Patient 2	Patient 3	Patient 4	Patient 5
Age	15	4	34	7	5
Sex	m	m	f	f	f
Country	Ukraine	Ukraine	Ukraine	Ukraine	Vietnam
Phenotype	Severe	Mild	Moderate	Moderate	Mild
Mimicking classic OI type	V	I	IV	IV	I
OI history in the family	No	Yes	Yes	No	No
Hyperplastic callus	Yes, extreme	Yes	Yes	No	Yes
Interosseous membrane calcification	Yes	Yes	Yes (fibula-tibia)	No	No
Radial head dislocation	Yes	Yes	Yes	No	Yes
Metaphyseal radiodense band	NA	Yes	Yes	No	Yes
Hearing loss	Yes, age 14	No	No	No	No
Dentinogenesis imperfecta	No, teeth brittle	No	No	No	
Sclera hue	Grayish	Bluish	Bluish	Grayish	Bluish
Number of fractures	8	4	26	16	5
Number of fractures per year	0.5	1	0.8	2.3	1
Time of the first fracture	1.3	7 months	at birth	3 months	2
First fractured bone	Femur	Left femur	Elbow, femur	Scapula	Lower leg
The most frequently fractured bone	Femur	Femur	Femur	Legs	Lower leg
Pyramidal/bell-shaped chest	Yes, severe	No	Yes	Yes	No
Scoliosis	Yes	No	Yes	Yes	No
Upper limb long bone deformity	Yes, mild	Yes, mild	Yes	No	Yes, mild
Lower limb long bone deformity	Yes	Yes, mild	Yes	Yes, mild	Yes, mild
Mobility	Immobile, lying	Walking	Walking (did not walk at age 9–14)	Walking	Walking
Height (Z score)	− 3.93	0.66	− 2.70	− 2.83	− 1.22
Birthweight and length	52/3450	51/2500	52/3300	50/2800	NA/2800
Joint laxity	No	Yes	Yes	No	NA

HPC and radial head dislocation was identified in 4/5 patients. Interosseous membrane calcification and metaphyseal radiodense bands were observed in 3/5 cases. Three patients had bluish and two had grayish eye sclera. There were no registered cases of DI among our patients. However, patient 1 developed teeth brittleness of an unknown nature. Patient 1 had also developed hearing loss, which started at the age of 14. Both patients 2 and 3, which came from family with a history of OI, had joint laxity. The overall number of fractures in patients 1–5 ranged from 0.5 to 2.3 per year. Patients developed mostly mild deformity of their long bones in both their upper and lower extremities. Scoliosis and chest deformity was found in 3/5 patients. Only one patient was immobile. According to their height Z scores, the patients are shorter than average in the age and sex-matched group. Growth retardation seemed to be greater with age (Table 1). Detailed descriptions of the patients’ cases are represented below.

### Patient 1

**Fig. 1:**
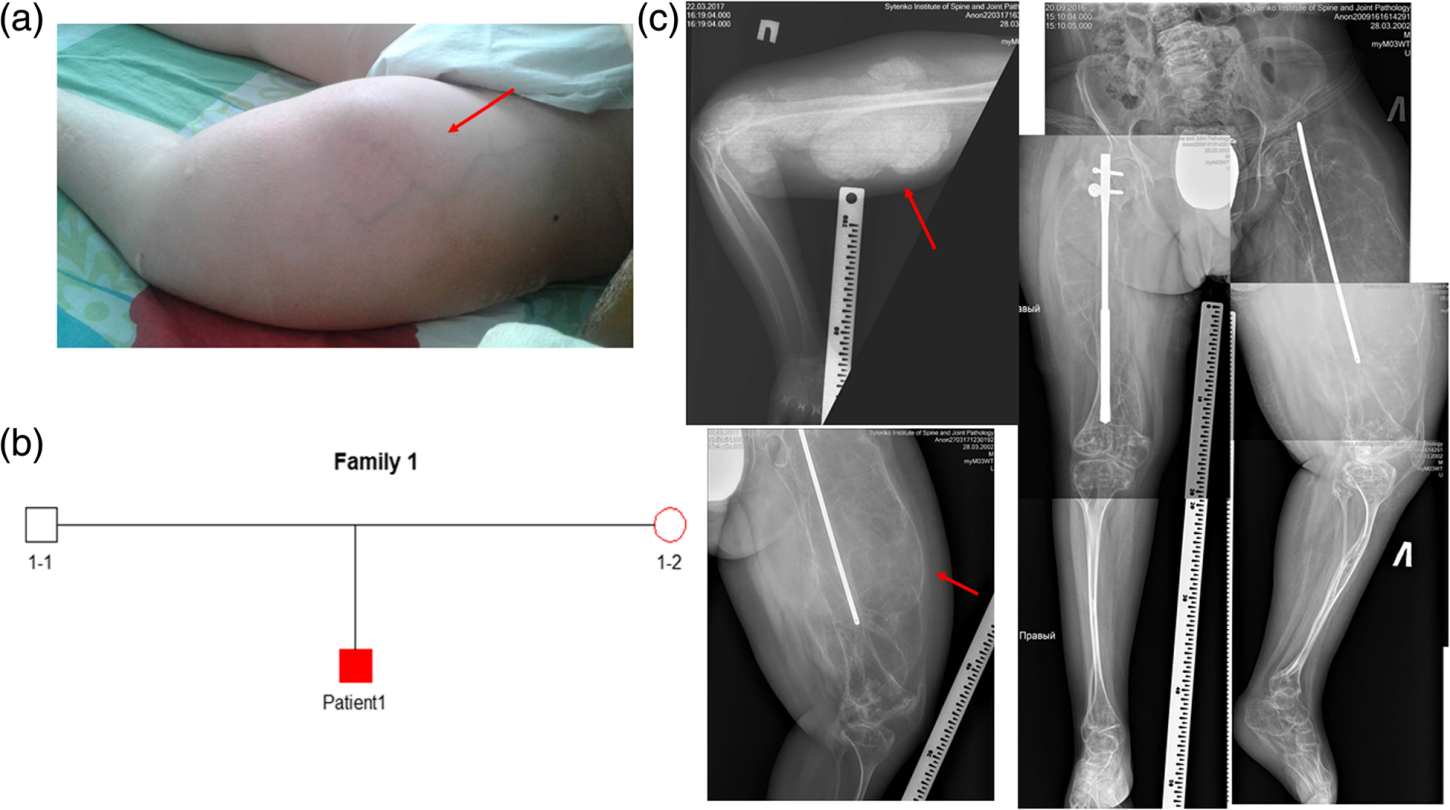
Patient 1. **a** Hyperplastic callus formation in the femur. **b** Genealogical tree of patient 1 (family 1). **c** Radiological features of patient 1

Patient 1 is a 15-year-old male (lock time September 2017) and the only child of a healthy non-consanguineous Ukrainian family (family 1) (Fig. 1b). His mother had her first pregnancy without a history of miscarriages. The pregnancy was full term without any health concerns. The patient’s birth weight was 3.5 kg (Z score − 0.06, 48% centile), his birth length was 52 cm (Z score 0.76, 78% centile), and he was in good condition immediately after birth. The patient’s current weight is 45 kg (Z score − 1.32, 9% centile) with a height of 135 cm (Z score − 3.93, 0% centile). The patient has grayish eye sclera, brittleness of teeth, and hearing loss, which started at the age of 14. The patient suffers from headaches, urolithiasis, and pyelonephritis.

The total number of fractures was eight. Patient 1 suffered his first fracture at the age of 1 year and 3 months in the right hip. The next fracture happened in the jaw, at the age of four, due to a fall. At the age of seven, the patient fractured his lower left leg. At the age of eight, the patient re-fractured their lower left leg along with fracturing their left arm. At the age of nine, the patient had received a blow to their hip, and within a month the patient had developed an irregular-shaped crack of the cortex where the blow had been dealt. The patient had equal thickening of the cortex on both side femurs. Within 2-months, a sarcoma-like ossification was discovered, sized 5 × 4 cm, without a clear contour line. After 1.5 months, the ossification enlarged to 7 × 5 cm with some thickening and a clearer contour line. In 2017, hyperplastic callus formation was extreme (Fig. 1c). The lower limb developed inflammatory symptoms: redness, fever, and enlarged in size (Fig. 1a). Patient 1 was diagnosed with pseudo osteosarcoma. Patient 1 underwent osteosynthesis. After a few months, the patient suffered a right hip fracture and underwent osteosynthesis of his right femur.

The patient developed deformation of the spine and combined deformities of his lower, upper limbs, and chest. The patient has HPC of the right humerus and both femurs (Fig. 1c), congenital synostosis of the right forearm (radioulnar interosseous membrane calcification), and contracture of his right elbow joint (radial head dislocation) (Fig. 1c). The patient suffers from severe HPC formation in the hips (from 2011, age 9). Due to severe callus, the patient is immobile and unable to sit (Fig. 1c). The bone mineral density (BMD) Z-score of the spine was − 5.9 and the lower arm received a Z-score BMD of − 3.6. The patient underwent bisphosphonate treatment with pamidronate.

Results of histological bone biopsy analysis showed that his callus bone tissue is immature and hypercellular. The patient’s new grown bone trabeculae have a high number of osteoblasts located at the basophil intercellular matter, with the inclusion of cartilage regions. Both the patient’s osteoblasts and osteocytes do not show any abnormalities. On the contour line, there is quite a significant number of osteoclasts. The intertrabecular space is filled with numerous blood vessels and fibroreticular tissue. The blood vessels are sinusoidal with large wide pores. The patient’s bone marrow is hypocellular and hydropic with regions with hemorrhagic infiltration.

### Patient 2

**Fig. 2:**
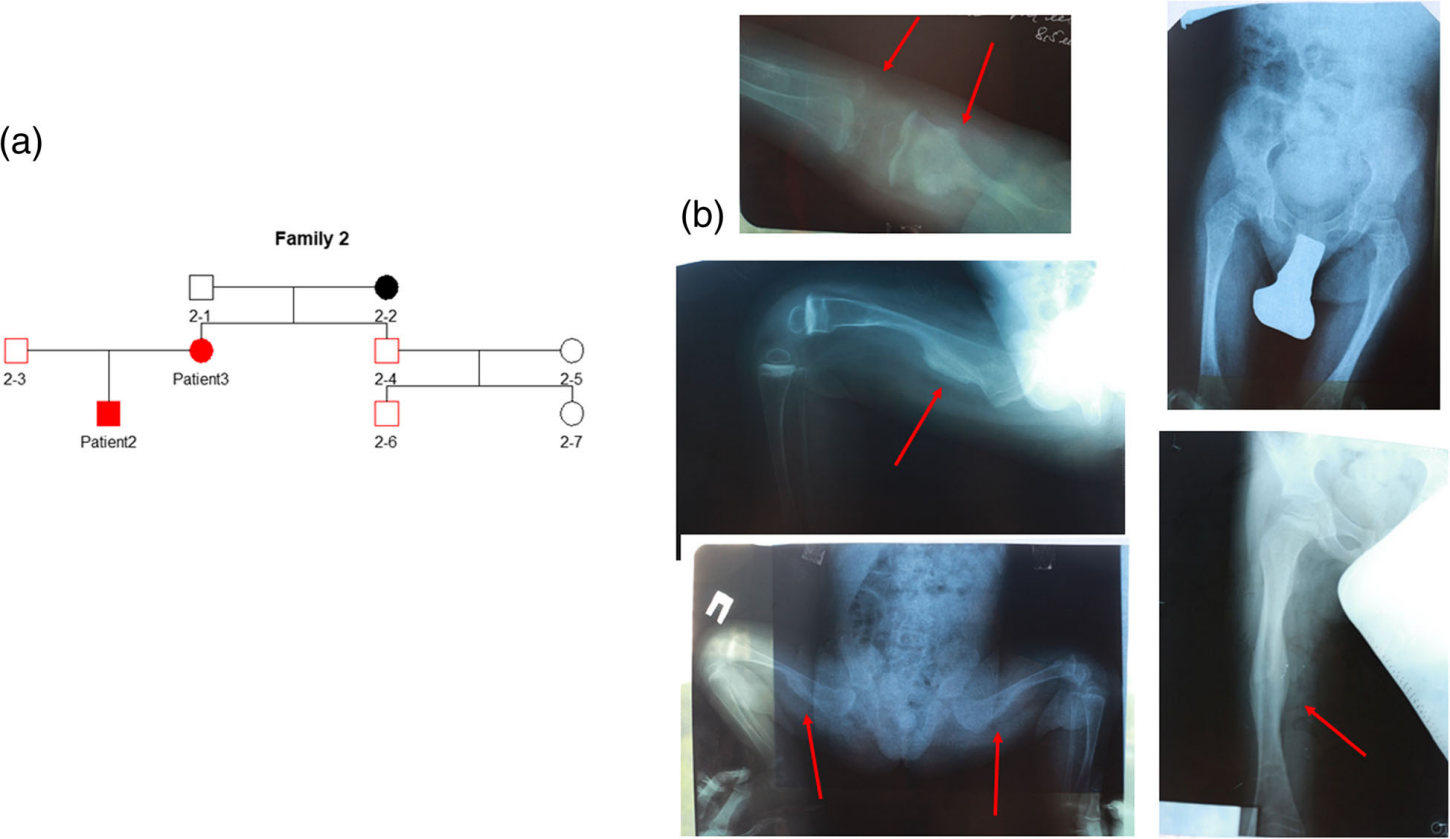
Patient 2. **a** Genealogical tree of patients 2 and 3 (family 2). **b** Radiological features of patient 2

Patient 2 is a 4-year-old boy (lock time May 2016) from a Ukrainian family with three generations of OI history from the mother’s line (family 2) (Fig. 2a). The mother had a healthy full-term pregnancy, without any previous miscarriages. His birthweight was 2.5 kg (Z score − 1.68, 5% centile), and his birth length was 51 cm (Z score 0.38, 65% centile). There were no signs of deformities or fractures after delivery.

The patient’s first fracture appeared at the age of 7 months in the femur during massage. At 8 and 11 months, the patient fractured both their right and left femur. Afterwards, the patient followed treatment with pamidronate. The last fracture happened at the age of 4, in the left forearm. The total number of fractures was 4.

The patient’s current weight is 15 kg (Z score − 0.68, 25% centile) and their height is 105 cm (Z score 0.66, 74% centile). The patient has bluish eye sclera and joint laxity. He is active and able to move independently. Signs of DI and hearing loss are absent.

Patient 2 has mild phenotype, mild deformities of chest, long lower and upper limb bones, with radial head dislocation and radioulnar interosseous membrane calcification (Fig. 2c). Investigation of X-rays showed the presence of HPC and a metaphyseal radiodense band (Fig. 2c).

### Patient 3

Patient 3 is a 34-year-old female from Ukraine (lock time May 2016) and the mother of patient 2 (family 2) (Fig. 2a). Her birthweight was 3.3 kg (Z score − 0.2, 42% centile), and her birth length was 52 cm (Z score 1.0, 85% centile). During being born, she had numerous fractures: both elbow, left hip, and both lower legs. The total number of fractures was 26. The majority of the fractures affected the lower limbs, especially the femur. She became immobile between the ages of 9 and 14.

**Fig. 3:**
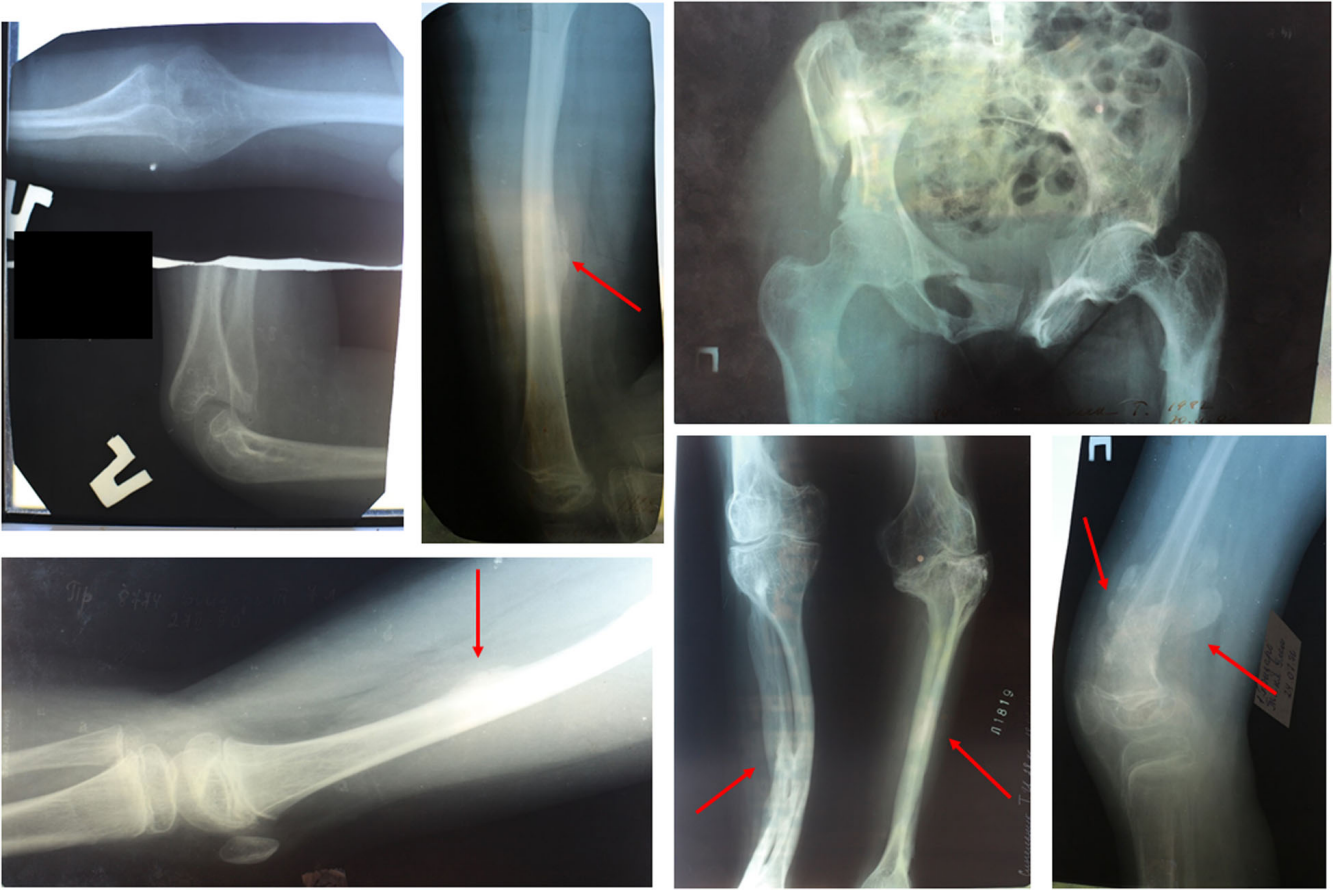
Patient 3. **a** Radiological features of patient 3

At the age of 9, the lower and upper limbs developed HPC. She also has calcification of the interosseous membrane in the fibula and tibia along with radial head dislocation. An investigation of X-rays revealed the presence of a metaphyseal radiodense band. The patient had chest deformation, scoliosis, and deformities of the long bones in both upper and lower limbs (Fig. 3a). The patient currently walks independently. The patient has moderate phenotype, mimicking OI type IV, no DI, or hearing loss. Her eye sclera is bluish. The patient has joint laxity. Her current weight is 42 kg and her height is 145 cm (Z score − 2.70, 0% centile).

### Patient 4

**Fig. 4:**
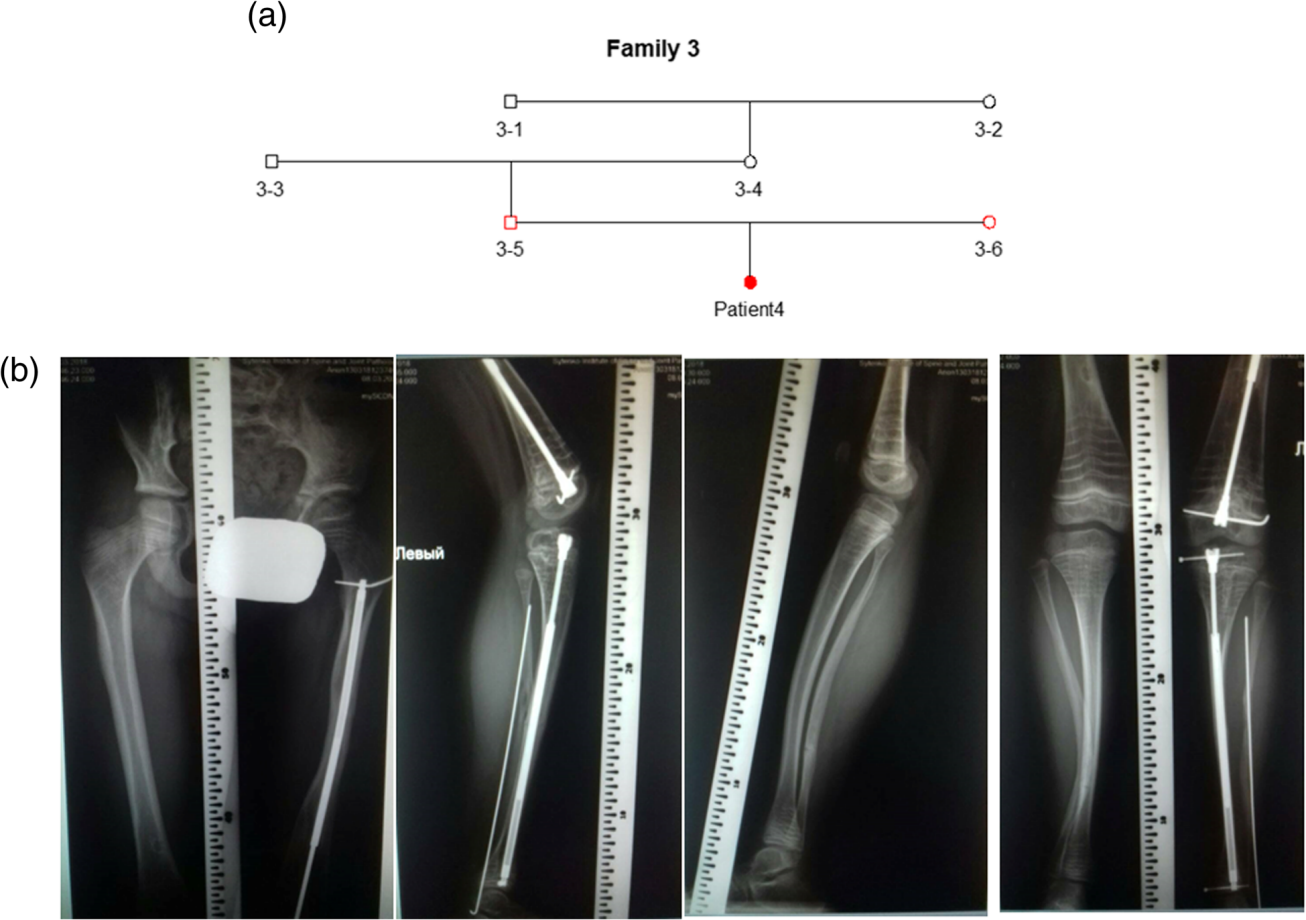
Patient 4. **a** Genealogical tree of patient 4 (family 3). **b** Radiological features of patient 4

Patient 4 is a 7-year-old girl from Ukraine (lock time May 2016). The patient has moderate OI phenotype, mimicking OI type IV without any visible features of OI type V. The patient was initially misdiagnosed with OI type IV. Patient 4 has no family history of OI (family 3) (Fig. 4a). Her birthweight was 2.8 kg (Z score − 1.18, 12% centile), and her birth length was 50 cm (Z score 0.28, 61% centile). There were no deformities or fractures during delivery; however, she had an unmineralized skull. At the age of 3 months, the first fracture appeared in the scapula during massage. The total number of fractures is 16. Most fractures affected the lower limbs. The patient’s left lower leg was fractured ten times. She had osteosynthesis on left lower and upper leg (Fig. 4b). The patient follows treatment with pamidronate. She has some deformities of lower limbs, spine, and chest (Fig. 4b). The patient had no hearing loss, DI, or joint laxity. Her eye sclera are gray. Her current weight is 17 kg (Z score − 2.22, 1% centile) and her height is 107 cm (Z score − 2.83, 0% centile). The patient is active and able to walk independently. Typical characteristics of OI type V, like HPC, interosseous membrane calcification, radial head dislocation, and a metaphyseal radiodense band, are absent.

### Patient 5

**Fig. 5:**
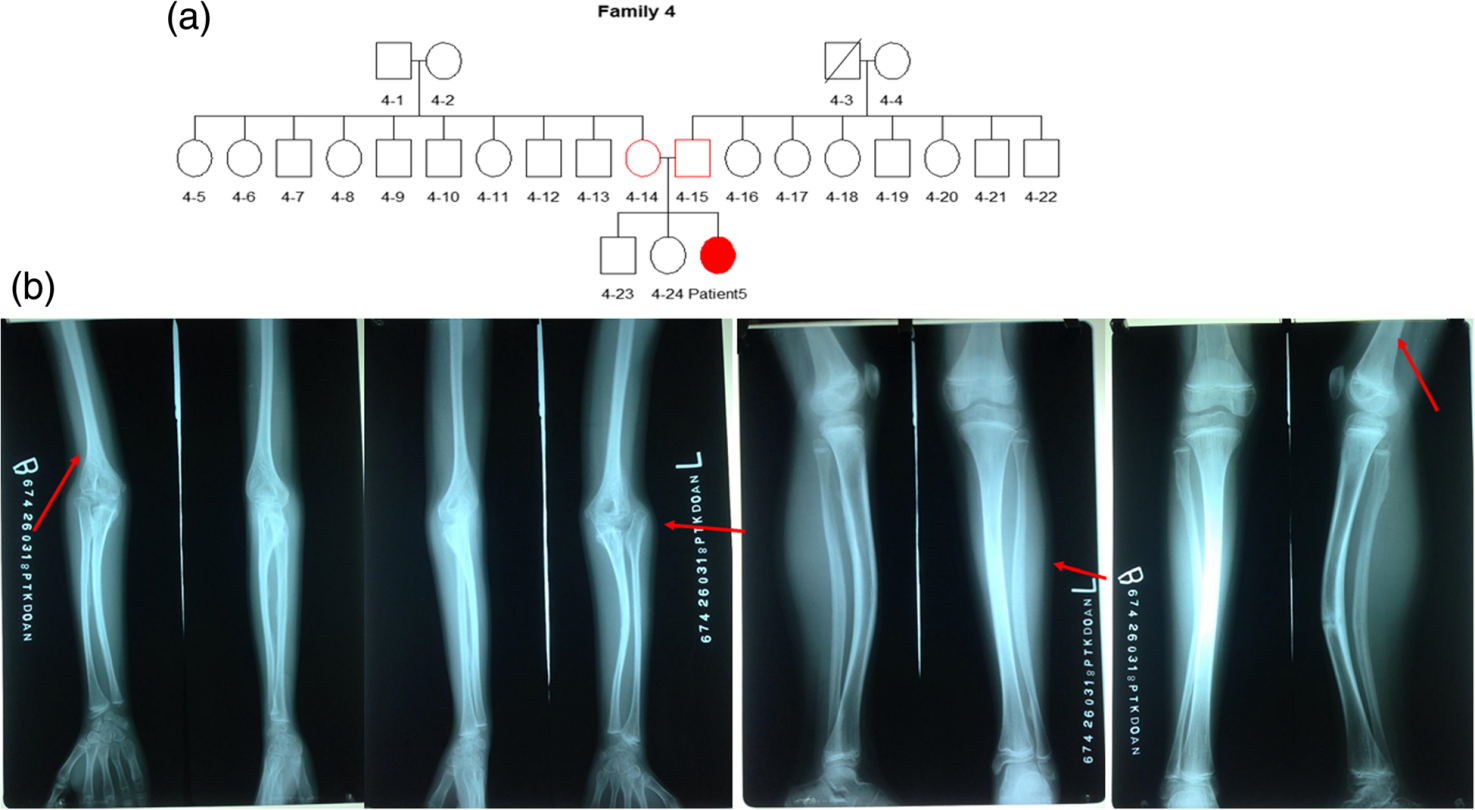
Patient 5. **a** Genealogical tree of patient 5 (family 4). **b** Radiological features of patient 5

Patient 5 is a 7-year-old girl from Vietnam, with mild OI phenotype (lock time 2015). Her father suffered some fractures in his forearms when he was under 10 years old. We do not have additional evidence of fractures in any of the other family members (family 4). An absence of consanguineous marriage was also confirmed (Fig. 5a). The mother had full-term pregnancy with 40 weeks of gestation, good health, and no history of miscarriages. Her birthweight was 2.8 kg (Z score − 1.18, 12% centile). Current clinical examination showed a weight 19 kg (Z score − 1.27, 10% centile) and height 115 cm (Z score − 1.22, 11% centile). She has blue sclera.

The patient suffered her first fracture at the age of 2 years old. The patient had five fractures on both side tibias. Mild deformities appeared in the long bones of the forearms and lower legs (Fig. 5b). She is able to move normally. The patient did not follow bisphosphonate treatment. On the radiological examination, ossifications of the interosseous membrane between the ulna and radius and congenital dislocation of the radial head were present (Fig. 5b).

## Discussion

Previous reports show that the proportion of OI type V patients may vary from 5 to 10% [10, 39]. According to our results, out of 337 OI patients in the UT OI biobank, less than 2% suffered from OI type V (five individuals). Interestingly, in a cohort of Palestinian OI patients, there were no carriers of the *IFITM5* pathogenic variants, as well as among our Estonian OI population [40]. We do not have evidence to assume, that such differences might come from distinctions in the population genetics of different ethnic groups. But rather, the difference may be due to differences in population size and recruitment of patients. We were also surprised to find a relatively higher number of type V patients among Ukrainian OI cohort as compared to our Vietnamese OI population, as sample sizes and methods of patient recruitment were comparable.

Both the de novo and familial nature of the *IFITM5* pathogenic variant was observed among our patients. In relation to previously published data, we could assume that the peak of the disease severity passes after puberty, and it does not affect reproduction in OI type V patients [25, 28, 41]. However, similarly to collagen-related OI, the majority of cases appear due to de novo pathogenic variants [42].

Both de novo and familial cases of type V showed a history of callus formation. As previous works have shown, the majority of patients had HPC [42]. Because of this, some patients may suffer from a series of misdiagnoses, which precede a proper diagnosis. For example, patient 1 from our current study was previously diagnosed with pseudo osteosarcoma due to extremely large HPC. Histological analysis has shown the presence of a mesh-like lamellation pattern typical for HPC in OI V [25]. The HPC in patient 1 represents the typical sign of OI V HPC, like primitive woven bone with inflammation, hypercellular trabeculae, and small cartilaginous islands [26].

Intriguing results were provided by study of Liu et al., which has shown that the typical OI type V pathogenic variant c.-14C > T in the *IFITM5* gene promoted apoptosis in osteosarcoma cells, inhibited tumor invasion, and promoted osteogenic differentiation [43]. However, the exact mechanism is not yet known, interconnections of osteosarcoma and OI type V in the form of a MALEP-IFITM5 look encouraging for the management of both diseases. Thus, patients with extreme cases of HPC might be of interest.

Calcification of interosseous membrane was previously detected in all OI type V patients older than age 4 [25, 44]. Similarly, to described Chinese OI patients, we do not have any evidence of interosseous membrane calcification in patient 4 and 5, who are 7 and 5 years old, respectively.

In addition to patterns of unusual mineralization, our patients suffered from ordinary OI symptoms, like bone fragility, recurrent fractures, skeletal deformations, hearing loss, joint laxity, teeth abnormalities, and bluish and grayish colored sclera. To our knowledge, hearing loss was identified in only one patient with OI type V [30]. Whereas, teeth abnormalities seemed to be more common. In the study of Kim et al., teeth abnormalities relevant to OI type V phenotype were identified in 11/16 patients [45].

All of these characteristics might be caused by the altered effect of mutated MALEP-IFITM5 protein on collagen type I, as decreases in collagen expression were previously identified in OI type V primary osteoblast cultures and transgenic mouse models of OI type V [35, 46]. Thus, type I and IV mimicking symptoms might be connected to the presence of a quantitative collagen defect in patients with OI type V. Whereas, the formation of enormous bone callus and interosseous membrane calcification might be caused by the effect of mutated MALEP-IFITM5 on the process of increased mineralization in the bone tissue.

Genetic testing ruled out misdiagnoses and confirmed OI type V in patient 1. It helped to build up an appropriate strategy for pharmacological and surgical treatment. Similarly, in the rest of revealed cases (patients 2–5), where classical OI was suspected, and treatment strategy was updated. More cautious treatment approach is needed to exclude risks and reduce potential complications, proceeding from the molecular defect of OI type V. Moreover, results can be further used to provide family planning counseling for these families and support cost-effective and rapid genetic diagnosis of affected family members in following generations.

Phenotype severity varied between patients in both OI type V symptoms and classical OI features. The number of fractures was different. As well as in other OI types, the occurrence of fractures seemed to decrease after puberty. However, growth retardation increased with age and seemed to correlate with phenotype severity. The phenotypical variation of OI type V has led us to the conclusion that despite the OI causing effect, the presence of a pathogenic variant in the *IFITM5* gene does not shape phenotype in its full guise. Additional modifying factors are not less important than the presence of the *IFITM5* pathogenic variant itself and are yet to be discovered. A deep phenotyping description of OI type V patients with different phenotype severity and the collection of their genetic material is required for the further investigation and understanding of OI type V.

## Conclusions

We have investigated the presence of the OI type V c.-14C > T 5′UTR *IFITM5* pathogenic variant in a cohort of OI patients negative for collagen I pathogenic variants with and without symptoms of OI type V. We have identified five individuals with OI type V from Ukrainian and Vietnamese origin with various phenotype severity. Patient 4 had no visible features of OI type V, whereas patient 1 had an extreme case of hyperplastic callus formation. Data from our patients supports the presence of classical OI features (hearing loss, bluish and grayish sclera, joint laxity, teeth abnormalities) in OI type V in addition to typical OI type V features such as abnormal mineralization. Due to high variation in clinical symptoms, we suggest the testing of *IFITM5* gene pathogenic variants in patients who lack OI type V features. Identifying what pathogenic variants cause OI is important for both doctors and patients, as it supports the organization of a proper treatment plan and follow ups for patient and helps for responsible family planning. Further investigations into the additional phenotype modifying factors and influence of MALEP-IFITM5 on osteogenesis should shed light on this rare and mysterious OI type.

## Data Availability

The datasets used and/or analyzed during the current study are available from the corresponding author upon reasonable request.

## Abbreviations

5′UTR: Five prime untranslated region
BMD: Bone mineral density
BRIL: Bone-restricted ifitm-like protein
DI: Dentinogenesis imperfecta
EDTA: Ethylenediaminetetraacetic acid
gDNA: Genomic DNA
HPC: Hyperplastic callus
MALEP: Five additional amino acids in the N-termini of the BRIL protein
OI: Osteogenesis Imperfecta
UT: University of Tartu
